# Colostrum-derived exosome based system: The next-generation nucleic acid delivery system with enhanced biosafety?

**DOI:** 10.1016/j.omtn.2022.10.013

**Published:** 2022-11-14

**Authors:** Jiayu Zhang, Hanqing Chen, Jun Chen

**Affiliations:** 1 CAS Key Laboratory for Biomedical Effects of Nanomaterials and Nanosafety, Multi-disciplinary Research Division, Institute of High Energy Physics and University of Chinese Academy of Sciences (UCAS), Chinese Academy of Sciences (CAS), Beijing 100049, P.R. China; 2 Department of Gastroenterology and Hepatology, Guangzhou Digestive Disease Center, Guangzhou First People's Hospital, School of Medicine, South China University of Technology, Guangzhou 510180, Guangdong, China

**Keywords:** milk/colostrum exosomes, exosome, siRNAs, pDNA, biosafety

Wallen et al. employed a colostrum exosome-based nanoparticle delivery system, exosome-PEI matrix (EPM), to help develop a flexible, testable therapeutic model system. Based on this system, plasmid DNA and small interfering RNA (siRNA) were delivered into *in vitro* cell lines and *in vivo*. Three essential severe acute respiratory syndrome coronavirus 2 (SARS-CoV-2) proteins—spike glycoprotein, nucleocapsid, and replicase—were expressed to mimic the expression of the viral genome; certain siRNAs were found to silence their matched proteins with 80%–95% efficiency, indicating their therapeutic potential. This technology reduces the requirement for biosafety levels (BSLs) in developing therapeutic siRNAs (especially for developing infectious disease drugs) and makes research simpler and more accessible.

The SARS-CoV-2 pandemic has highlighted the need for nucleic-acid-based gene therapy due to the lack of viable drugs to treat SARS-CoV-2 infection. Two main barriers currently limit nucleic acid drug discovery: first, limited biosafety level 3 (BSL3) containment facilities; and second, rapid mutation of the virus often renders vaccines ineffective and requires rapid development of new vaccines as a response. siRNAs have expanded the field of gene therapy by inhibiting the expression of target proteins. However, barriers in the application of siRNAs include the fact that unprotected siRNAs are not readily taken up by cells and that the presence of nucleases *in vivo* is sufficient to pose a significant threat to the integrity of siRNA duplexes. Therefore, siRNA can only provide effective therapeutic intervention if it reaches the target site through a suitable delivery system. Liposomes are currently the most applied siRNA transfection reagent, and liposomal siRNA preparations have entered clinical trials, including therapies targeting hypercholesterolemia, transthyretin-mediated amyloidosis, and cancer.^1^ In the study of these different systems, several agents have emerged that are particularly effective for siRNA delivery, including the use of cationic or ionizable lipids, shielding lipids, cholesterol, and targeting ligands.^1^

In this paper, to achieve rapid and efficient screening of therapeutic siRNAs, Wallen et al. built a model system using milk/colostrum exosomes. First, polyethylenimine (PEI) was shown to be required for efficient plasmid DNA binding to exosomes, and cytotoxicity assays demonstrated that PEI incorporation did not cause significant cytotoxicity. Second, three SARS-CoV-2 antigen proteins were expressed: S1 protein, N protein, and RdRp. Multiple siRNA duplexes were then designed based on the plasmid sequences of each viral antigen. These siRNA duplexes and the viral antigen expression plasmids were loaded separately in EPM and delivered. By analyzing the expression of viral antigens, the authors screened for efficient siRNAs. These experiments demonstrate that the system established by the authors is capable of screening therapeutic siRNAs at low safety levels. The authors also demonstrated that the addition of lactoferrin (LF) as a targeting ligand enhanced the response of this model system.

The authors consider the highlights of their work to be the ability to screen for therapeutic siRNAs at low BSLs and the fact that plasmid DNA (pDNA) and siRNAs can be delivered in one system. However, other delivery systems have similar characteristics, and this appears to be a credit to molecular biology. For example, liposome-based systems can also deliver plasmids expressing viral proteins and the corresponding siRNAs without the need for high BSL isolation sites.^2^ Therefore, there is no compelling reason for EPM to possess significant advantages in these aspects.

The best part of the study, in my opinion, is the use of a colostrum exosome-based delivery system. Although nucleic acid drugs have demonstrated benefits in infectious disease prevention, cancer treatment, etc., public concerns about vaccine safety persist, and some of these concerns stem from nucleic acid vectors. A colostrum-derived nucleic acid delivery system would go some way to alleviating public concerns. Morever, colostrum is available in large quantities, and the start material is less restricted. Additionally, some molecular markers on the surface of exosomes may be able to reduce the phagocytosis of exosomes by macrophages and prolong the *in vivo* circulation time of the delivery system.^3^

Another interesting point is that *in vivo* assays using EPM delivery of spike proteins and nucleocapsids produced antibodies against viral antigens within 45 days of target delivery. These findings seem to suggest that the EPM delivery system could be used as a nucleic acid vaccine vector for the treatment of infectious or other diseases. Unfortunately, the article does not explore this further.

Overall, this study is interesting and achieves the authors’ goal of flexible screening of therapeutic siRNAs at low safety biological levels. However, the most important feature, which was the successful use of the system as a nucleic acid vaccine vector, was not explored further. Therefore, one can only expect them to publish more studies in the area.
